# Invasive pulmonary aspergillosis among patients with severe community-acquired pneumonia and influenza in ICUs: a retrospective cohort study

**DOI:** 10.1186/s41479-024-00129-9

**Published:** 2024-05-25

**Authors:** Wei-Chun Lee, Che-Chia Chang, Meng-Chin Ho, Chieh-Mo Lin, Shaw-Woei Leu, Chin-Kuo Lin, Yu-Hung Fang, Shu-Yi Huang, Yu-Ching Lin, Min-Chun Chuang, Tsung-Ming Yang, Ming-Szu Hung, Yen-Li Chou, Ying-Huang Tsai, Meng-Jer Hsieh

**Affiliations:** 1 Department of Pulmonary and Critical Care Medicine, Chang-Gung Medical Foundation, Linkou Chang-Gung Memorial Hospital, No.5, Fuxing St., Guishan Dist., Taoyuan, 333 Taiwan (ROC); 2 Department of Respiratory Therapy, School of Medicine, Chang-Gung University, Taoyuan, Taiwan; 3 Department of Pulmonary and Critical Care Medicine, Chiayi Chang-Gung Memorial Hospital, Chang-Gung Medical Foundation, Chiayi, Taiwan; 4 Department of Respiratory Care, Chang Gung University of Science and Technology, Chiayi, Taiwan

**Keywords:** Community-acquired pneumonia, Invasive pulmonary aspergillosis, Critical care, Prevalence, Mortality, Diagnostic test

## Abstract

**Rationale:**

The prevalence, clinical characteristics, and outcomes of invasive pulmonary aspergillosis in patients with severe community-acquired pneumonia (CAP) in intensive care units remain underestimated because of the lack of a disease-recognition scheme and the inadequacy of diagnostic tests.

**Objectives:**

To identify the prevalence, risk factors, and outcomes of severe CAP complicated with invasive pulmonary aspergillosis (IPA) in intensive care units (ICUs).

**Methods:**

We conducted a retrospective cohort study including recruited 311 ICU-hospitalized patients with severe CAP without influenza or with influenza. Bronchoalveolar lavage fluid (BALF) samples were from all patients and subjected to mycological testing. Patients were categorized as having proven or probable Aspergillus infection using a modified form of the AspICU algorithm comprising clinical, radiological, and mycological criteria.

**Measurements and main results:**

Of the 252 patients with severe CAP and 59 influenza patients evaluated, 24 met the diagnostic criteria for proven or probable Aspergillus infection in the CAP group and 9 patients in the influenza group, giving estimated prevalence values of 9.5% and 15.3%, respectively. COPD and the use of inhaled corticosteroids were independent risk factors for IPA. IPA in patients with severe CAP was significantly associated with the duration of mechanical support, the length of ICU stay, and the 28-day mortality.

**Conclusions:**

An aggressive diagnostic approach for IPA patients with severe CAP and not only influenza or COVID-19 should be pursued. Further randomized controlled trials need to evaluate the timing, safety, and efficacy of antifungal therapy in reducing IPA incidence and improving clinical outcomes.

## Introduction

Invasive pulmonary aspergillosis (IPA) in the intensive care unit (ICU) can be prevalent and deadly. Despite a growing understanding of invasive fungal infections, IPA remains difficult to diagnose and treat, partly due to the underlying diseases with which this opportunistic infection is associated [1]. The reported incidence of IPA in the ICU is as high as 19%, with substantial variation across institutions and patient subsets [2, 3]. Early treatment is a determinant of the overall survival from IPA, which is problematic in groups where the suspicion of infection is low. This difficulty may contribute to the > 80% mortality rate due to IPA in the ICU [4, 5].

The definitions of invasive fungal diseases were updated in 2019 by the European organization for research and treatment of cancer and the mycoses study group education and research consortium (EORTC/MSGERC) for use in the research setting and in severely immunocompromised patients [6]. However, IPA can affect ICU-hospitalized patients who may not have classic host risk factors. Some patient groups that would be typically considered immunocompetent are also at high risk of IPA [7, 8]. According to one large retrospective cohort study, among patients with influenza who are admitted to ICUs, 19% developed invasive aspergillosis and 44% lacked classic host risk factors [2]. Other examples are male sex, smoking history, the use of corticosteroids and/or immunobiological therapies, prolonged stay in the ICU, and chronic obstructive pulmonary disease (COPD) [9–14]. Recently, SARS-CoV-2 infection (COVID-19) has also been reported to be associated with pulmonary aspergillosis, and the incidence varies widely across studies (0%–33%) [15]. The features of IPA in this setting differ substantially from those in neutropenic patients. Therefore, the AspICU algorithm and the modified AspICU algorithm were developed to guide the investigation of patients with Aspergillus infection from clinical, radiological, and mycological criteria [2, 9].

Additionally, despite advances made in diagnosis and antimicrobial therapy, CAP remains an important cause of morbidity and mortality, especially in patients requiring hospitalization. However, due to the absence of a disease-recognition scheme and limited access to sensitive tests, the prevalence, risk factors, and clinical outcomes of severe CAP-complicated IPA among patients with respiratory failure who are admitted to ICUs are underdetermined. Identifying the actual prevalence and risk factors may help reveal better strategies for the prevention and early recognition of severe CAP-complicated IPA and identify patients requiring intensive care to mitigate the negative outcomes of severe CAP with associated IPA. Therefore, this study aimed to review the causes of severe CAP complicated with IPA in ICU-hospitalized patients and identify risk factors for ICU stay and mortality in these patients.

## Materials and methods

### Study design and participants

The study is a retrospective cohort study of ICU-hospitalized patients treated from August 2015 through July 2019 at our institution in Chiayi, Taiwan. The study protocol was approved by the institutional review board of the Chang Gung Memorial Hospital, Taiwan. We included a study cohort of patients with severe CAP without influenza and a control group of patients with severe CAP and associated influenza. Patients with severe CAP were admitted to the ICU from outside the hospital with respiratory insufficiency or unstable vital signs due to pneumonia.

Inclusion criteria: All patients were more than 18 years old and were admitted to the ICU for more than 24 h with acute respiratory failure. They had new or progressive pulmonary infiltrates on imaging. The definition of severe CAP was based on the American Thoracic Society/Infectious Diseases Society of America guidelines [16]. The inclusion criterion for patients with severe CAP with influenza was a confirmed influenza infection based on a positive airway polymerase chain reaction test (PCR; QiAamp Viral RNA Mini Kit, TAIGEN Bioscience Corporation, Taiwan). All patients received prior antibiotics when they were admitted to the ICU, and underwent standardized bronchoalveolar lavage (BAL) sampling that was available for mycological testing within the first 3 days after they were admitted to the ICU. The galactomannan (GM) enzyme immunoassay on BALF samples was performed using the Platelia Aspergillus enzyme immunoassay per the manufacturer’s instructions. Other microorganisms were also identified from blood and bronchoscopy cultures.

We excluded patients who had hospital-acquired pneumonia as defined in the American Thoracic Society/Infectious Diseases Society of America guidelines [17], those in whom respiratory failure was not the primary reason for ICU admission, those with insufficient available information, those with a history of IPA, and those with a confirmed alternative diagnosis lasting until the end of the follow-up period. We also excluded all patients in whom the only mycological evidence for IPA was a positive culture from the lower respiratory tract (sputum, tracheal aspirate) for Aspergillus species but who had a negative or unavailable BAL culture or galactomannan test, these patients were considered colonized.

The definition of probable IPA was modified from the AspICU algorithm based on the presence of clinical, radiological, and mycological criteria [2]. Patients with proven IPA as documented by histopathology or direct microscopic evidence of necrotic lung tissue with angioinvasive by acute angle branching septate fungal hyphae or cultures positive for *Aspergillus spp*. from lung tissue specimens. Patients with severe CAP with or without influenza were reviewed, and a consensus was achieved to ascertain that the modified IPA definition was met.

### Risk factors and clinical outcomes

Clinical outcomes of interest included the duration of mechanical ventilator support, the duration of ICU stay and hospital stay, and mortality (28-day, ICU, and 90-day). Risk factors for aspergillosis were assessed, including age, sex, history of TB or other microbial infections, inhale or oral steroid use (all patient with COPD or asthma had used inhaled corticosteroids > 1 year), comorbidities including hypertension, diabetes, liver cirrhosis, chronic kidney disease, chronic obstructive pulmonary disease (all patient with COPD had spirometry data with post-bronchodilator FEV1/FVC < 0.7), asthma, cancer malignancy, hematologic malignancy, AIDS, and autoimmune disease), Acute Physiology and Chronic Health Evaluation (APACHE) II scores [18], and the concurrent diagnosis of influenza. Besides, we also records the coinfection of IPA in the severe CAP and influenza groups.

### Statistical analysis

The results are presented as frequencies and percentages, mean values with standard deviations, or medians with interquartile ranges unless otherwise indicated. Student’s t-test was to perform comparisons between normally-distributed continuous variables while the Mann–Whitney U test was used for these comparisons between variables with skewed data distributions. The Chi-square test and Fisher's exact test were used for categorical variables. Risk factors for the 28-day mortality and 90-day mortality among these patients were evaluated using univariate and multivariate generalized linear regression analyses. Variables showing statistical significance in univariate analyses (*p* < 0.05) were included in the multivariate logistic regression analysis using the backward elimination method. The 95% confidence intervals of all comparisons are also reported. A two-tailed *p* value of < 0.05 was considered statistically significant. All statistical analyses were conducted using the Statistical Package for the Social Sciences statistical software (version 26.0; SPSS Inc, Chicago, IL, USA).

## Results

### Patient characteristics of the severe CAP and influenza groups

**Fig. 1:**
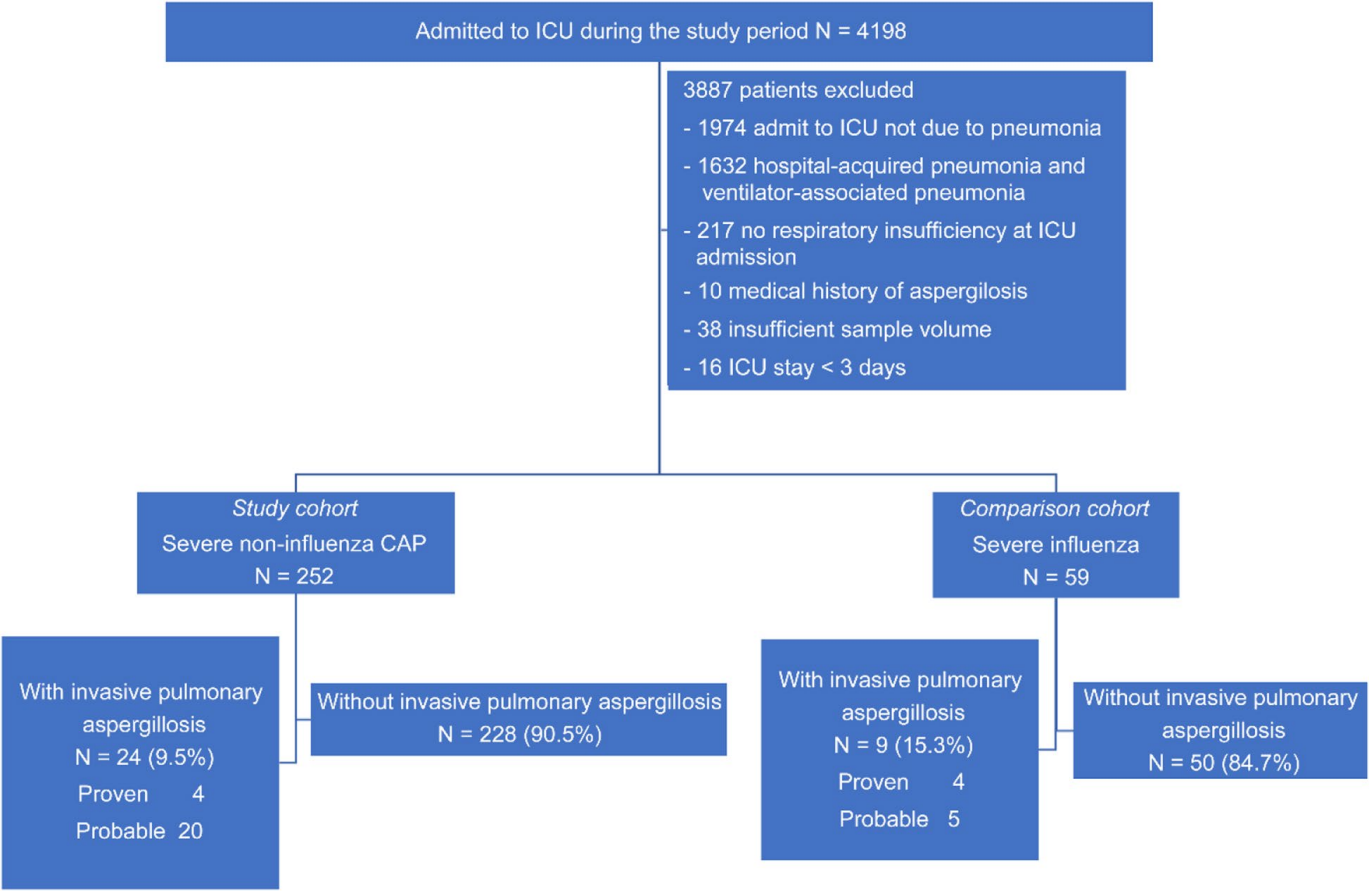
Distribution of patients with severe non-influenza community-acquired pneumonitis and severe influenza pneumonitis

Three hundred and eleven patients (252 ICU-hospitalized patients with severe CAP as the study cohort and 59 ICU-hospitalized patients with severe influenza as the comparison cohort) were eligible for this study (Fig. 1). Table 1 summarizes the baseline characteristics of the patients in the two groups. Compared to patients in the influenza group, significantly more ICU-hospitalized patients with severe CAP had a personal history of oral corticosteroid use (4% and 0%, respectively; *p* < 0.005), active cancer malignancy (13% and 5%, respectively; *p* < 0.05), autoimmune diseases (4% and 0%, respectively; *p* < 0.005), older age (mean values: 71.94 and 62.32, respectively; *p* < 0.001), lower body mass index (BMI) (mean values: 22.36 and 24.55, respectively; *p* < 0.005), and higher APACHE II scores on admission (mean values: 20.49 and 18.85, respectively; *p* < 0.005). Besides, acute kidney injury was significantly more prevalent among patients with severe CAP (57% and 24%, respectively; *p* < 0.001), and thromboembolism events were significantly less common among these patients (6% and 17%, respectively; *p* < 0.05).

**Table 1:** Demographic data of patients with severe community-acquired pneumonitis or severe influenza infection in the ICUs

	Severe CAP *n* = 252	Severe influenza *n* = 59	*P* value
Gender (M/F)	165/87	29/30	0.157
Age (year, Mean ± SD)	71.94 ± 14.24	62.32 ± 16.88	< 0.001^*^
BMI (Mean ± SD)	22.36 ± 4.88	24.55 ± 4.80	0.002^*^
APACHI II score (Mean ± SD)	20.49 ± 6.19	18.58 ± 5.62	0.003^*^
Use of MV, N (%)	247 (98%)	54(91.5%)	0.089
Use of ECMO, N (%)	1 (0.4%)	4 (7%)	0.06
ARDS	146 (58%)	28 (47%)	0.145
Acute kidney injury	143 (57%)	14 (24%)	< 0.001^*^
Thromboembolism events	16(6%)	10(17%)	0.044^*^
Invasive pulmonary aspergillosis, N (%)	24 (9.5%)	9 (15.3%)	0.262
- Proven, N	4	4	
- Probable, N	20	5	
- GM > 1 on BALF alone, N	19	7	
- GM > 0.5 on serum alone, N	1	0	
- GM positive of both BALF and serum, N	4	2	
- BALF GM Threshold OD(Mean ± SD)	3.78 ± 1.98	4.02 ± 2.02	
1–1.5, N	4	1	
1.5–3, N	5	2	
> 3, N	14	6	
MV days (Mean ± SD)	20.21 ± 19.18	19.31 ± 19.7	0.747
ICU days (Mean ± SD)	19.21 ± 13.4	16.31 ± 9.43	0.054
Hospital days (Mean ± SD)	35.35 ± 28.14	31.24 ± 21.97	0.295
28-day mortality, N (%)	54 (21%)	12 (20%)	0.637
ICU mortality, N (%)	63 (25%)	11 (19%)	0.453
90-day mortality, N (%)	87(35%)	14 (24%)	0.092
*Co-morbidities, N (%)*			
Cardiovascular history	78 (31%)	13 (22%)	0.152
Hypertension	123 (49%)	32 (54%)	0.455
COPD	30 (12%)	7 (12%)	0.985
Asthma	9 (4%)	1 (2%)	0.464
ICS 28 days before ICU	10 (4%)	2 (3%)	0.836
OCS 28 days before ICU	9 (4%)	0 (0%)	0.003^*^
TB	8 (3%)	1 (2%)	0.543
Liver cirrhosis	27 (11%)	7 (12%)	0.800
Diabetes mellitus	87 (35%)	25 (42%)	0.301
Chronic kidney disease	27 (11%)	9 (15%)	0.328
Advanced cancer malignancy	33 (13%)	3 (5%)	0.027^*^
Autoimmune disease	11 (4%)	0 (0%)	0.001^*^
AIDS	1 (0.4%)	0 (0%)	0.629

*CAP* community acquired pneumonia, *BMI* body mass index, *APACHE II* Acute Physiology and Chronic Health Evaluation II score, *ARDS* acute respiratory distress syndrome, *ECMO* Extracorporeal Membrane Oxygenation, *MV* mechanical ventilation, *COPD* chronic obstructive pulmonary disease, *ICS* inhaled corticosteroid, *OCS* oral corticosteroid, *TB* tuberculosis, *AIDS* Acquired immunodeficiency syndrome

^*^ *P* < 0.05, Chi-Square test, Mann–Whitney U test

### Prevalence of IPA

Twenty-five patients in the severe CAP group and nine patients in the influenza group met the clinical, radiological, and mycological diagnostic criteria for IPA, giving an estimated prevalence of Aspergillus infection of 9.5% and 15.3%, respectively (*p* = 0.262).

### Patient characteristics and clinical outcomes of IPA in the severe CAP and influenza groups

**Fig. 2:**
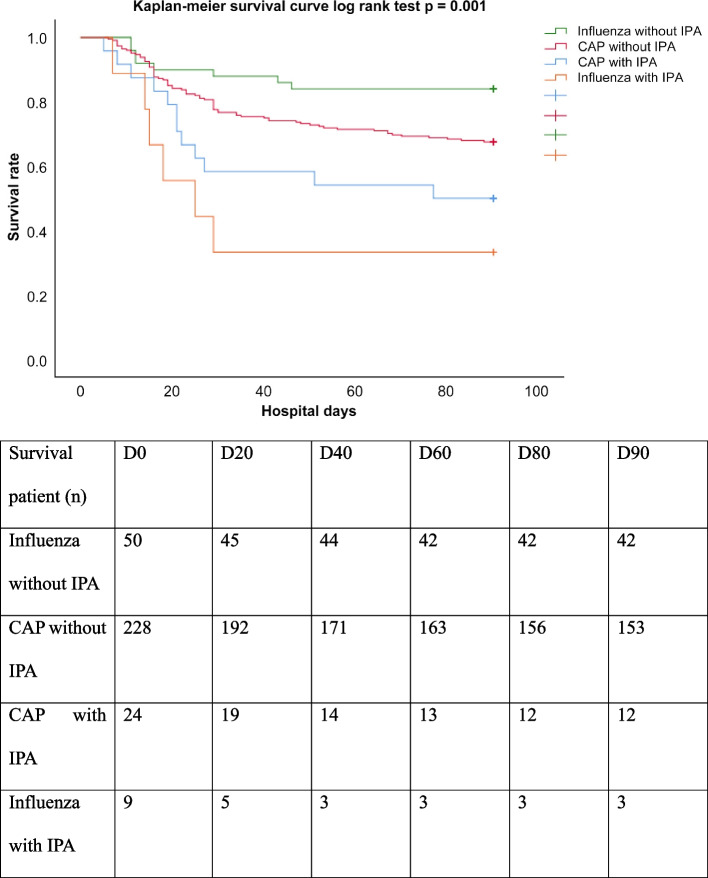
Kaplan–Meier survival curve

Table 2 summarizes the baseline characteristics of patients with or without pulmonary aspergillosis in the severe CAP and influenza groups. Among patients with severe CAP, those in the IPA group had significantly higher APACHE II scores upon admission (mean values: 23.6 and 20.2, respectively; *p* < 0.05), longer durations of mechanical support (mean values: 27.9 days and 19.4 days, respectively; *p* < 0.05), longer ICU stay (mean values: 29.8 days and 18.1 days, respectively; *p* < 0.05), and higher 28-day mortality (42% and 19%, respectively; *p* < 0.05). By contrast, in the influenza group, patients with pulmonary aspergillosis had significantly shorter durations of mechanical support (mean values: 11.8 days and 20.7 days, respectively; *p* < 0.05), shorter hospital stay (mean values: 18.4 days and 33.5 days, respectively; *p* < 0.05), higher 28-day mortality (67% and 10%, respectively; *p* < 0.05), higher ICU mortality (67% and 12%, respectively; *p* < 0.05), and higher 90-day mortality (67% and 16%, respectively; *p* < 0.05). Figure 2 shows that the Kaplan–Meier survival curves for the four division groups continue to diverge throughout the 90-day follow-up. The curves are significantly different per the log-rank test (*p* = 0.001).

**Table 2:** Demographic data of patients with or without invasive pulmonary aspergillosis infection

	**Severe CAP, ** ***n*** ** = 252**			**Severe influenza, ** ***n*** ** = 59**		
	**With IPA** ***n*** ** = 24**	**Without IPA** ***n*** ** = 228**	***p*** **-value**	**With IPA** ***n*** ** = 9**	**Without IPA** ***n*** ** = 50**	***p*** **-value**
Age, mean ± SD	74.58 ± 12.18	71.67 ± 14.43	0.341	60.98 ± 11.58	71.93 ± 17.42	0.152
Gender, n (%)			0.874			0.033^*^
Male	17 (70.8%)	148 (58.7%)		8 (88.9%)	21 (42%)	
Female	7 (29.2%)	80 (413%)		1 (11.1%)	29 (58%)	
BMI (kg/m^2^)	21.3 ± 5.2	22.47 ± 4.84	0.264	21.96 ± 3.29	25.02 ± 4.90	0.077
APACHE II score, mean ± SD	23.63 ± 5.45	20.16 ± 6.18	0.009*	17.44 ± 3.71	18.78 ± 5.898	0.516
Use of MV, N (%)	24 (100%)	224(98.2%)	0.466	8 (88.9%)	46(92%)	0.763
Use of ECMO, N (%)	0 (0%)	1 (0.4%)	0.746	2 (22%)	2 (4%)	0.256
ARDS	18 (75%)	128 (56%)	0.59	4 (44%)	24 (48%)	0.847
Acute kidney injury	17 (71%)	126 (55%)	0.132	1 (11%)	13 (26%)	0.263
Thromboembolism events	2(8%)	14(6%)	0.677	1(11%)	9(18%)	0.619
MV days (Mean ± SD)	27.88 ± 22.54	19.40 ± 18.67	0.039*	11.78 ± 7.29	20.66 ± 20.94	0.026^*^
ICU days (Mean ± SD)	29.79 ± 22.01	18.09 ± 11.69	0.017*	14.22 ± 6.2	16.68 ± 9.90	0.476
Hospital days (Mean ± SD)	40.04 ± 30.55	34.86 ± 27.90	0.391	18.44 ± 7.78	33.54 ± 22.93	0.001^*^
28-day mortality, N (%	10 (42%)	44 (19%)	0.045*	6 (67%)	5 (10%)	0.009^*^
ICU mortality, N (%)	10 (42%)	53 (23%)	0.095	6 (67%)	6 (12%)	0.011^*^
90-day mortality, N (%)	12 (50%)	75 (33%)	0.094	6 (67%)	8 (16%)	0.016^*^
*Co-morbidities,* N (%)						
Cardiovascular history	10 (42%)	68 (30%)	0.234	1 (11%)	12 (24%)	0.327
Hypertension	11 (46%)	112 (49%)	0.760	4 (44%)	28 (56%)	0.530
COPD	5 (21%)	25 (11%)	0.270	4 (44%)	3 (6%)	0.062
Asthma	2 (8%)	7 (3%)	0.379	0 (0%)	1 (2%)	0.675
ICS 28 days before ICU	3 (13%)	7 (3%)	0.190	1 (11%)	1 (2%)	0.442
OCS 28 days before ICU	2 (8%)	7 (3%)	0.379	0 (0%)	0 (0%)	1.000
TB	1 (4%)	7 (3%)	0.772	0 (0%)	1 (2%)	0.675
Liver cirrhosis	4 (17%)	23 (10%)	0.324	1 (11%)	6 (12%)	0.941
Diabetes mellitus	9 (37%)	78 (34%)	0.748	5 (56%)	20 (40%)	0.552
Chronic kidney disease	3 (12%)	24 (10%)	0.767	2 (22%)	7 (14%)	0.536
Advanced cancer malignancy	5 (21%)	28 (12%)	0.337	1 (11%)	2 (4%)	0.380
Autoimmune disease	0 (0%)	11 (5%)	0.001*	0 (0%)	0 (0%)	1.000
AIDS	0 (0%)	1 (0.4%)	0.629	0 (0%)	0 (0%)	1.000

*CAP* community acquired pneumonia, *BMI* body mass index, *APACHE II* Acute Physiology and Chronic Health Evaluation II score, *ARDS* acute respiratory distress syndrome, *ECMO* Extracorporeal Membrane Oxygenation, *MV* mechanical ventilation, *COPD* chronic obstructive pulmonary disease, *ICS* inhaled corticosteroid, *OCS* oral corticosteroid, *TB* tuberculosis, *AIDS* Acquired immunodeficiency syndrome

^*^P < 0.05, Chi-Square test, Mann–Whitney U test

### Risk factors for IPA

The findings in Table 3 illustrate the multivariate logistic regression analysis of risk factors contributing to IPA among patients with severe CAP with or without influenza. COPD (OR:2.993, 95% CI = 1.232–7.276; *p* < 0.05) and the use of inhaled corticosteroids (OR:3.782, 95% CI = 1.009–14.177; *p* < 0.05) were significant independent risk factors for IPA in all patients. On the contrary, influenza infection was not an independent risk factor for IPA (OR:0.568, 95% CI = 0.243–1.326; *p* = 0.191). Multivariate logistic regression analyses in each group revealed that the use of inhaled corticosteroids (OR:4.333, 95% CI = 1.010–18.585; *p* < 0.05) was a significant independent risk factor for IPA in the severe CAP group. Besides, the presence of COPD comorbidities was the only independent risk factor for IPA in the influenza group (OR:11.294, 95% CI = 1.883–88.435; *p* < 0.05).

**Table 3:** Multivariate logistic regression of the risk factors for invasive pulmonary aspergillosis in patients with severe non-influenza CAP or severe influenza

Factors	Risk for invasive pulmonary aspergillosis		
	OR	95% CI	*P* value
*All patients*			
Influenza vs. non-influenza CAP	0.568	0.243–1.326	0.191
COPD	2.993	1.232–7.276	0.016^*^
ICS 28 days before ICU	3.782	1.009–14.177	0.048^*^
*Severe CAP patients*			
COPD	1.764	0.580–5.364	0.317
ICS 28 days before ICU	4.333	1.010–18.585	0.048^*^
*Severe influenza pneumonitis patients*			
COPD	11.294	1.883–67.733	0.008^*^
ICS 28 days before ICU	2.883	0.094–88.435	0.544

*OR* Odds ratio, *95% CI* 95% confidence interval, *CAP* community acquired pneumonia, *COPD* chronic obstructive pulmonary disease, *ICS* inhaled corticosteroid

^*^ *P* < 0.05

### Risk factors for 28-day mortality and 90-day mortality

Table 4 presents the risk factors for 28-day mortality and 90-day mortality. Multivariate logistic regression analyses revealed that acute respiratory distress syndrome (ARDS), acute kidney injury, and a history of active advanced cancer events were risk factors for 28-day mortality and 90-day mortality in all patients (all *p* < 0.05). IPA affected the 28-day mortality (OR:4.167, 95% CI = 1.686–10.297; *p* < 0.05), and the development of thromboembolism affected the 90-day mortality (OR:3.295, 95% CI = 1.188–9.135; *p* < 0.05) in all studied patients. In the severe CAP group, IPA (OR:2.987, 95% CI = 1.244–7.710; *p* < 0.05) and acute kidney injury (OR:2.973, 95% CI = 1.310–6.750; *p* < 0.05) were independent risk factors for 28-day mortality. Meanwhile, acute respiratory distress syndrome was the dependent risk factor for both 28-day mortality (OR:21.438, 95% CI = 5.016–91.632; *p* < 0.05) and 90-day mortality (OR:10.474, 95% CI = 4.684–22.554; *p* < 0.05) in the severe CAP group. On the other hand, in the influenza group, IPA (OR:63.303, 95% CI = 5.649–709.317; *p* < 0.05, and OR:81.691, 95% CI = 6.084–1096.868; *p* < 0.05, respectively) and acute kidney injury (OR:2.973, 95% CI = 1.310–6.750; *p* < 0.05, and OR:21.962, 95% CI = 2.147–224.695; *p* < 0.05, respectively) were independent risk factors for both 28-day mortality and 90-day mortality. The occurrence of thromboembolism events was the independent risk factor for 90-day mortality (OR:23.809, 95% CI = 2.226–254.690; *p* < 0.05) in the influenza group.

**Table 4:** Multivariate logistic regression of the risk factors for 28-day, ICU, and 90-day mortality in patients with severe non-influenza CAP or severe influenza

**1. 28-day mortality**			
	28-day mortality		
	OR	95% CI	*P* value
*All patients*			
Invasive pulmonary aspergillosis	4.167	1.686–10.297	0.002^*^
Acute kidney injury	2.936	1.444–5.968	0.003^*^
Acute respiratory distress syndrome	9.078	3.402–24.225	< 0.001^*^
Advanced cancer malignancy	2.513	1.073–5.883	0.034^*^
*Severe CAP patients*			
Invasive pulmonary aspergillosis	2.987	1.244–7.710	0.014^*^
Acute kidney injury	2.973	1.310–6.750	0.009^*^
Acute respiratory distress syndrome	21.438	5.016–91.632	< 0.001^*^
*Severe influenza pneumonitis patients*			
Invasive pulmonary aspergillosis	63.303	5.649–709.317	0.001^*^
Acute kidney injury	16.781	1.171–165.001	0.016^*^
**2. 90-day mortality**			
	90-day mortality		
	OR	95% CI	*P* value
*All patients*			
Acute kidney injury	2.417	1.310–4.459	0.005^*^
Acute respiratory distress syndrome	6.834	3.390–13.778	< 0.001^*^
Thromboembolism events	3.295	1.188–9.135	0.022^*^
Advanced cancer malignancy	2.435	1.055–5.621	0.037^*^
*Severe CAP patients*			
Acute respiratory distress syndrome	10.474	4.684–22.554	< 0.001^*^
*Severe influenza pneumonitis patients*			
Invasive pulmonary aspergillosis	81.691	6.084–1096.868	0.001^*^
Acute kidney injury	21.962	2.147–224.695	0.009^*^
Thromboembolism events	23.809	2.226–254.690	0.009^*^

*OR* Odds ratio, *95% CI* 95% confidence interval, *CAP* community-acquired pneumonitis

^*^P < 0.05

### Co-infection of IPA in the severe CAP and influenza groups

The most common coinfection pathogens of IPA in the severe CAP and influenza were *Klebsiella pneumonia, Escherichia coli,* and *Pseudomonas aeruginosa, that* were presented on Table 5.

**Table 5:** Co-infection in the groups of CAP with IPA and influenza with IPA

Co-infection	Influenza with IPA *N* = 9	CAP with IPA *N* = 24
*Klebsiella pneumonia*	1	5
*Methicillin-resistant Staphylococcus aureus (MRSA)*	1	1
*Pseudomonas aeruginosa*	1	3
*Escherichia coli*	1	4
*Serratia marcescens*	1	0
*Enterobacter cloacae*	0	1
*Group B Streptococcus*	0	2
*Neisseria species*	0	1
*Cytomegalovirus*	1	1

*CAP* community acquired pneumonia, *IPA* invasive pulmonary aspergillosis

## Discussion

Our study expanded on the results of previous studies and provided further evidence that the presence of COPD comorbidities and inhaled corticosteroid use are independent risk factors for IPA. Indeed, of 252 patients without influenza infections admitted to the ICU with severe CAP, the presence of COPD comorbidities and inhaled corticosteroid use increased the risk of IPA by 2.993-fold and 3.782-fold, respectively. The length of mechanical ventilation use, ICU stay, and 28-day mortality were significantly higher among these patients with IPA. Diagnosing IPA is challenging; so, patients who have severe CAP with comorbid COPD or a history of inhaled corticosteroid use in the ICU might have an even higher incidence of IPA and worse 28-day mortality.

In addition to hosts with the already-mentioned chronic conditions and comorbidities, patients admitted to ICUs with respiratory tract infections have a significantly increased risk of IPA [19, 20]. It is most likely a result of respiratory epithelial disruption and aberrant host immune responses [21]. The inflammatory homeostasis at the alveolus is disrupted during infection, with proinflammatory cytokines produced, leading to local tissue disruption, allowing the translocation of *Aspergillus spp.* to the tissues and bloodstream [22], and causing aspergillosis. The reported incidence of IPA in patients who are critically ill varies widely, from less than 1% to 6.9% [11, 23, 24]. One study of 2901 patients with severe influenza in the ICU found co-infection in 17% of patients, of which *Aspergillus spp*. accounted for 7% [25]. Geographical locations revealed a similar IPA prevalence among hospitalized patients with influenza between European (10%) and non‐European (11%) studies, and the IPA prevalence in the subset of nine studies using the modified AspICU criteria was 13% [26]. Our study revealed a 15.3% prevalence of IPA in patients who have severe CAP with influenza, which is similar to the findings of previous studies conducted in Taiwan [27]. On the contrary, the largest retrospective cohort study reports a 5% prevalence of IPA in 315 non-immunocompromised patients admitted to the ICU with CAP [2]. However, BAL sampling was done only in 55% of these patients, and the BAL galactomannan test was performed only in 34% of patients in the severe CAP group; thus, the actual prevalence of IPA might have been underestimated. In our study, all ICU-hospitalized patients with severe CAP and respiratory failure underwent bronchoscopy within three days per our routine clinical practice. Therefore, the 9.5% prevalence estimated in our study is higher than expected based on the findings of previous studies and might be more representative of the real-life situation. The slightly increased prevalence in the influenza group and the absence of a significant difference in prevalence between this group and the CAP group in our study highlights the importance of routine bronchoscopy with BAL sampling evaluation, a possibility clinicians should be aware of and which should be addressed in studies of large populations. 62% patient of or study experienced transient desaturation, tachycardia, and tachypnea when performing bronchoscope with bronchoalveolar lavage, and all of them recovered 1 to 2 h later.

In our study, IPA was proven by histopathologic evidence in 4 out of 24 patients (17%) in the CAP group and 4 out of 9 patients (44%) in the influenza group. Histopathological evidence of the presence of *Aspergillus spp.* from the tracheobronchial tree or alveolar tissue remains the gold standard for the diagnosis of IPA. However, due to the risk of worsening hypoxia and coagulopathy, sampling of lung tissues in ICU-hospitalized patients is rarely performed. Therefore, the galactomannan optical index in BALF and serum has been recommended as an alternative entry route to better identify patients with aspergillosis [28]. The OD cutoff above which a BAL galactomannan test should be considered positive is still a subject of debate. D’Haese and colleagues demonstrated that an OD cuff value of 1.0 in BALF offers a 79.7% sensitivity and 93.8% specificity for IPA [29]. They also presented that the use of an extremely stringent OD threshold of 3.0 for the BALF GM index has been reported to have 56% sensitivity and 100% specificity, which is likely to yield an underestimate. When using the OD threshold of 3.0 in our study, the minimal prevalence was 5.6% in the CAP cohort and 10% in the influenza cohort. This is because the true prevalence of IPA remains uncertain, given the variation range of BALF GM threshold values.

Several risk factors for invasive aspergillosis in ICU-hospitalized patients were well-established in previous studies. In our study, we found that ICU patients with COPD comorbidities and inhaled corticosteroid use had a 2.99-fold risk and 3.78-fold risk, respectively, of IPA. Further, the use of inhaled corticosteroids was the independent risk factor in the CAP group, and the presence of COPD was the independent risk factor in the influenza group, respectively. Several studies [3, 11, 30–32] also have reported that patients with severe COPD are at risk of IPA. COPD may increase susceptibility to IPA for several reasons, including structural changes in lung architecture, the prolonged use of corticosteroids, frequent hospitalization, broad-spectrum antibiotic treatment, invasive procedures, mucosal lesions and impaired mucociliary clearance, and comorbid illnesses such as diabetes mellitus, alcoholism, and malnutrition. It is also possible that abnormalities or deficiencies in surfactant proteins, alveolar macrophages, and Toll-like receptors play a role in the pathogenesis of IPA in some patients with COPD [33, 34]. Besides, inhaled corticosteroids are commonly administered to asthmatic and COPD patients. Previous studies reported that corticosteroids impair monocyte, macrophage, and neutrophil phagocytosis of *Aspergillus fumigatus* [35]. One study found that the mycobiome was highly varied, with severe asthmatics receiving corticosteroids carrying higher loads of fungi, with the most common ones being the *Aspergillus fumigatus* complex [36]. Similar results were also reported among COPD patients based on sensitive culture techniques and using high-dose ICS [37]. When the alveolus is disrupted during severe CAP infection, allowing the translocation of *Aspergillus spp.* to the tissues and the bloodstream, and leading to IPA. Therefore, in patients with severe CAP with the associated use of inhaled corticosteroids and COPD, clinicians should beware of the likelihood of IPA developing and the need for aggressive diagnostic workup and treatment of positive cases. On the contrary, unlike other studies, influenza infection was not a dependent risk factor for IPA (Table 3) in our study. A possible explanation was the higher prevalence of IPA in the non-influenza severe CAP group. There is no doubt that influenza is a well-established risk factor for IPA. However, the risk of developing IPA in severe CAP patients, especially when complicated by respiratory failure and the need for ventilator support as mentioned in our study, might be underdetermined. Therefore, we reiterate the importance of routine bronchoscopy with BAL sampling evaluation, especially for patients with severe CAP and those who require ventilation support.

Mortality for IPA patients remains high, with the need for improved diagnosis and treatment in this complex and challenging disease. A previous study reported an in-hospital mortality rate of 46% among patients with IPA [1]. In Taiwan, Sun et al. [38] reported, in a series of 407 IPA patients from 2002 to 2012, that the overall case fatality rate was > 30%. Our study found that a significantly longer duration of mechanical support, longer ICU stay, and higher 28-day mortality among patients having severe CAP with IPA. By contrast, in the influenza group, patients with IPA had significantly higher 28-day mortality, higher ICU mortality, and higher 90-day mortality. Figure 2 shows that the Kaplan–Meier survival curves for the four division groups continue to diverge throughout the 90-day follow-up, indicating significantly lower values in the CAP with IPA group and the influenza with IPA group. The development of acute kidney injury and acute respiratory distress syndrome in ICU-hospitalized patients was well recognized as an independent risk factor for short-term and long-term mortality [39–41]. In addition to that, IPA causes a 2.987-fold increase in the 28-day mortality of ICU-hospitalized patients with severe CAP in our study (Table 4-1). Similar results were also reported among intubated patients with SARS-CoV-2 or influenza pneumonia [42]. Though the development of ARDS was an independent risk factor for 90-day mortality in the severe CAP group (Table 4-2), patients with IPA may contribute to developing ARDS that is difficult to manage, leading to worsened outcomes [2, 43].

Although all patients with IPA received antifungal therapy after probable or proven IPA was diagnosed, high early mortality was reported in our study. Therefore, the early administration of antifungal therapy in critically ill patients with invasive aspergillosis may reduce the incidence of IPA, reduce the associated mortality, and improve clinical outcomes [44]. However, two recent studies demonstrated that antifungal prophylaxis did not improve the survival of COVID-19- or influenza-associated pulmonary aspergillosis in critically ill patients [45]. Further large-scale randomized controlled trials are warranted to evaluate the timing, efficacy, and safety of antifungal therapy with respect to IPA incidence and clinical outcomes.

Our study has some limitations. First, given the nonrandomized retrospective design of this study, confounding cannot be ruled out, and a standardized diagnostic approach toward IPA was not used in all patients. The retrospective analysis may introduce bias from incomplete information. Strengths of the study include the fact that all our patients underwent standard bronchoscopy and BALF collection within 3 days after admission, leading to a complete laboratory dataset, which strengthened our data analysis and reduced the influence of hospital-acquired or ventilator-associated IPA to a minimum. Secondly, the study was carried out in a single center; therefore, the extrapolation of our findings to small primary care ICUs should be done with caution. The prevalence of IPA in patients having severe CAP with influenza in our study is similar to that reported in previous studies in other parts of Taiwan, which provides the relay incidence of IPA in the CAP group.

The prevalence of IPA was higher than prior recognition among patients with severe CAP in the ICU. IPA was independently associated with COPD comorbidities and the use of inhaled corticosteroids. IPA was independently associated with the duration of mechanical support, the length of ICU stay, and the 28-day mortality. Therefore, an aggressive diagnostic approach for IPA patients with severe CAP and other critical illnesses, not only influenza or COVID-19, should be pursued. Besides, further RCTs also need to evaluate the timing, safety, and efficacy of antifungal therapy to reduce IPA incidence and improve clinical outcomes.

## Data Availability

No datasets were generated or analysed during the current study.
